# Assessment of dental caries among a group of institutionalized orphan children compared to parented school children: case–control study

**DOI:** 10.1186/s12903-023-02915-1

**Published:** 2023-04-05

**Authors:** Nagwa Mohamed Ali Khattab, Mennat Allah Ashraf Abd-Elsabour

**Affiliations:** 1 Professor of Pediatric Dentistry and Dental Public Health, Faculty of Dentistry Ain Shams University, Cairo, Egypt; 2 Assistant Lecturer, Pediatric & Community Dentistry Department, Faculty of Dentistry, Ahram Canadian University, Giza, Egypt

**Keywords:** Dental caries, Orphan children, Governmental orphanages, Non-governmental orphanages

## Abstract

**Background:**

It has been well documented that the absence of family support influences the general and oral health of children. Literature regarding the oral health status of institutionalized orphan children, who lost their families' support, especially in Egypt, remains vague. Therefore, the current study was carried out to assess dental caries among two groups of institutionalized orphan children, and compare their results with a group of parented school children in Giza, Egypt.

**Methods:**

A total of 156 children were included in this study, residing in a non-governmental orphanage, a governmental orphanage, and parented children attending private primary school. Written informed consent was obtained before the start of the study from the legal guardians or the child's parent. The dental examination was carried out as recommended by the WHO. DMF and def indices were used to assess dental caries for primary and permanent teeth. Also, the unmet treatment needs index, care index, and significant caries index were calculated.

**Results:**

The results revealed that mean values for DMF total score were 1.86 ± 2.96, 1.80 ± 2.54, and 0.7 5 ± 1.29 for, non-governmental, governmental orphanages, and school children respectively. While the mean def total scores were 1.69 ± 2.58, 0.41 ± 0.89, and 0.85 ± 1.79 for non-governmental, governmental orphanages, and school children, respectively. There was a high level of unmet treatment needs, especially among orphans. The significant caries index was 2.5, 4.29, and 2.17 for, non-governmental, governmental orphanages, and school children, respectively.

**Conclusions:**

Within the limitation of this case–control study, the institutionalized orphanage children had a high prevalence of dental caries and worse caries experience compared to parented school children. Effective oral health preventive strategies are required to improve the oral health status and oral health practices of those children.

**Trial registration:**

The trial was registered on ClinicalTrial.gov (ID: NCT05652231).

## Introduction

Family support and care are influencing factors in the child's well-being and health, including his oral and dental health [1]. The child tends to imitate his parents in the essential health practice, and his caries experience is influenced by his caregiver's cultural knowledge and attitude towards the oral health [2–4]. Prevention of dental caries relies on a properly balanced diet and good oral hygiene practice, and both should be supervised by a responsible guardian, to allow the child to enjoy decent dental health and well-being [5]. Seeking professional preventive measures and early treatment of dental caries in children is another important family role in keeping the child’s dental health [6, 7].

UNICEF defined an orphan as a child who has lost one or both of his parents or has been deserted by them before the age of eighteen [8, 9]. Orphans, therefore, are considered deprived and socially handicapped populations, whose disease liability is high [8]. Most orphan children are raised in orphanage institutions, which provide them with housing shelter, social, educational, and medical care. The compromised socioeconomic status of the institutionalized orphans, along with overcrowding, low care-taker to child ratio, poor nutrition, lack of adequate psychological support, lack of adequate facilities, and underestimation of the importance of dental treatment by the orphanages care-takers, all are contributory factors that compromise the oral health status of these deprived children [10–12].

Institutionalized orphans have shown a compromised oral health status in terms of dental caries, dental trauma, dental pain experience, and oral hygiene status, in comparison to their parented counterpart children [13–16].

Oral health education programs and dental health services are recommended by the WHO to be planned in accordance with the survey studies, that throw light on the oral health status of the community in interest [17]. Although many descriptive studies were run to estimate the prevalence of dental caries in different communities and populations, there are inadequate studies were conducted to reflect light on the orphan children communities, especially on Egyptian orphans [13–16, 18–21].

It is assumed that parental care is an essential factor in maintaining the child’s oral health and well-being, which includes dental caries experience. When the child is deprived of his parents' shelter and care, his entire well-being is expected to be compromised, and subsequently his oral health. To the best of our knowledge, dental caries experience in institutionalized orphan children, who are deprived of family care, in comparison to the children who are sheltered by their parents in Egypt remains vague. This study aimed to assess dental caries experience in two groups of Egyptian institutionalized orphan children, resident in two orphanages, compared to a matched group of parented school children.

## Subjects and methods

### Study design

This study was an observational case–control study, in which the study sample was collected from three randomly selected settings, non-governmental, governmental orphanages, and a privet primary school, from which parented children were recruited as a control group. All children were examined for dental caries experience, and the results of the three groups were analyzed. The recruitment took a place during the last two weeks of November 2022. This study is reported according to the Strengthening the Reporting of Observational Studies in Epidemiology (STROBE) Statement [22].

### Ethical approval and trial registration

Ethical approval for the research protocol was obtained from Institutional Review Board Organization IORG0010868, Faculty of Oral & Dental Medicine, Ahram Canadian University (ACU). Research Number: IRB00012891. This study was performed per the ethical standards laid down in the Declaration of Helsinki. The trial was registered on ClinicalTrial.gov (ID: NCT05652231).

### Sample size estimation

A power analysis was designed to have adequate power to apply a statistical test of the null hypothesis that there is no difference would be found between the study groups regarding the assessment of dental caries experience. By adopting an alpha level of (0.05), a beta of (0.2) (i.e., power = 80%), and an effect size (f) of (0.32) calculated based on the results of a previous study; [13] the predicted sample size (n) was a minimal of 52 children in each of the three study groups, i.e. 156 children in total. Sample size calculation was performed using G*Power version 3.1.9.7.

### Authorities’ approval and ethical regulations

Local authorities were reached, and their approval was obtained to enter the selected orphanages and private primary school. The headmasters of the orphanages and the school were contacted, the aim of the study was explained to them, and their approval was obtained.

The Parents'/ guardians’ acceptance was obtained through informed consents, laid down by Institutional Review Board Organization IORG0010868, Faculty of Oral & Dental Medicine, Ahram Canadian University, that were sent through the school to all parents of eligible children to take their signed consent to examine their children. Only children whose parents accepted to participate in the study were included in the control group. For orphan children, informed consents were signed by the children’s guardian/ orphanage authorities. The child's accent was also obtained from all participants in the target populations. All children who required dental care were referred to ACU Pediatric Dental Clinic, in which they were provided free-of-charge high-quality dental care.

### Participants

The selected population was recruited from the orphanages or the school, in Giza, Egypt. *Inclusion criteria* were cooperative, apparently healthy children, aged from 6 to 12 years old, with no history of chronic illness or medication, and their responsible guardian accept to participate in the study. *Exclusion criteria* were children with enamel/dentine developmental defects. All eligible children who presented on the examination days had the same opportunity to participate in the study. lists of non-governmental, governmental orphanages and private primary schools in Giza were obtained, and one non-governmental orphanage and another governmental orphanage in addition to one private primary school were selected randomly by lottery, then 52 children were recruited from each of the three institutions.

### Grouping

Control group (private primary school): included 52 children attending primary private school.

Study group 1 (non-governmental orphanage group): included 52 children who are residents in a non-governmental orphanage.

Study group 2: (governmental orphanage group): included 52 children who are residents in a governmental orphanage.

### Examination procedure

Before conducting the examination, inter-examiner and intra-examiner calibration were established for both investigators, by repeatedly examining children attending ACU Pediatric Dental Clinic, and reliability was tested and was proved to be within the accepted range (Kapa test = 0.89).

Examination of the study children was carried out on an ordinary chair, under normal daylight, using mirrors, probes, tweezers, cotton rolls, and personal protective barriers, following the criteria and methods recommended by WHO [23].

### Data collection

Demographic data, medical history, and dental history were recorded in an administrative chart for each study participant, along with four simple questions exploring their brushing habits, snacking habits, and visits to the dentists, and their answers were obtained by interviewing each participant. Then the child was examined for dental caries prevalence, and caries experience was recorded using def and DMF, or DMF indices [24–26]. The restorative care index [27] and the unmet treatment needs index [28] were calculated for each child. Then, the significant caries index was calculated for the three study groups.

### Data management and statistical analysis

Data entry and analysis were performed using the Statistical Program Statistical Package for the Social Sciences (SPSS) version 20. Descriptive statistics and the categorical data were analyzed using the Chi-Square test and Fisher exact test. Mann–Whitney U-test for comparing the mean/standard deviation (SD) of DMFT and deft for the control and study groups. The level of significance was set at 5%.

## Results

**Fig. 1:**
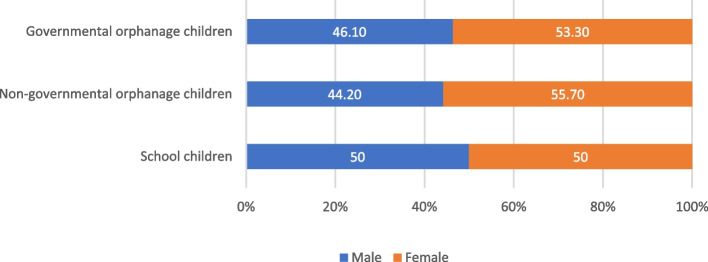
Distribution of the children in different study groups according to sex

A total of 156 children (52 /group) were included in the study. The total number of examined males and females was 73 (46.8%), and 83(53.2), respectively. The demographic characteristics of all participants are demonstrated in Table 1, and Fig. 1.

**Table 1:** Demographic characteristics of the study population

Variables	Primary Private school (Control group)	Non-Governmental orphanage (Study Group)	Governmental orphanage (Study Group)	*p*-value*
	***N*** **(%)**	***N*** **(%)**	***N*** **(%)**	
**Gender**				
Male	26 (50)	23 (44.20)	24 (46.10)	0.83
Female	26 (50)	29 (55.70)	28 (53.30)	
Total	52 (100)	52 (100)	52 (100)	––
**Age**				
6—< 9	22 (42.3)	27 (51.9)	23 (44.2)	0.85
9 -12	30 (57.6)	25 (48.1)	29 (55.7)	
Total	52 (100)	52 (100)	52 (100)	––-

^*^p-value: Chi Square Test, *p* ≤ 0.05 considered significant

**Fig. 2:**
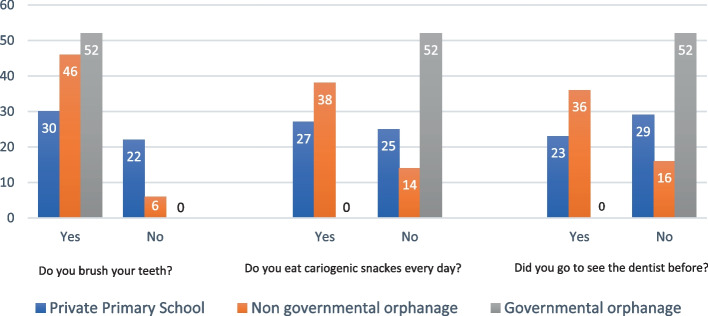
Frequency of responses of the study groups children to questions about their dental health care

Participants' answers to each of the questions revealing their oral care habits are demonstrated in Table 2, and Fig.2.

**Table 2:** Frequency (N) and percentage (%) of responses of the study participants to questions about their dental health care

Question	Private primary school (Control group) *N* (%)	Non-governmental orphanage (Study group 1) *N* (%)	Governmental Orphanage (Study group 2) *N* (%)	*p*-value*
Do you brush your teeth?				0.0^d^
Yes	30 (57.7)	46 (88.5)	52 (100)	
No	22 (42.3)	6 (11.5)	0 (0)	
Frequency of tooth brushing				0.0001^d^
No	22 (42.3)	6 (11.5)	0 (0)	
Irregular	9 (17.3)	11 (21.2)	8 (15.4)	
Once/day	4 (7.7)	9 (17.3)	36 (69.2)	
Twice/day	10 (19.2)	16 (30.8)	8 (15.4)	
More than twice/day	7 (13.5)	10 (19.2)	0 (0)	
Do you eat cariogenic snacks every day?				0.0^d^
Yes	27 (51.9)	38 (73)	0 (0)	
No	25 (48)	14 (26.9)	52 (100)	
Did you go to see the dentist before?				0.0^d^
Yes	23 (44.2	36 (69.2)	0 (0)	
No	29 (55.8)	16 (30.8)	52 (100)	

*p-value: Fisher Exact Test, ^a^*p* ≤ 0.05, ^b^*p* ≤ 0.01, ^c^*p* ≤ 0.001and.^d^*p* ≤ .0001

**Fig. 3:**
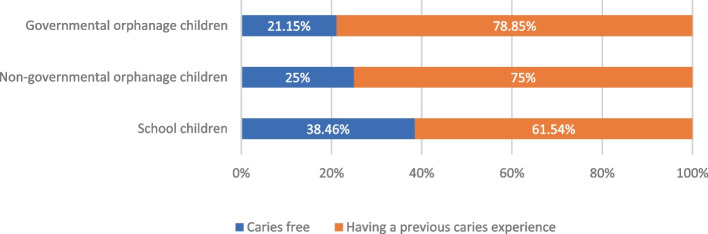
Prevalence of dental caries among the different study groups

**Fig. 4:**
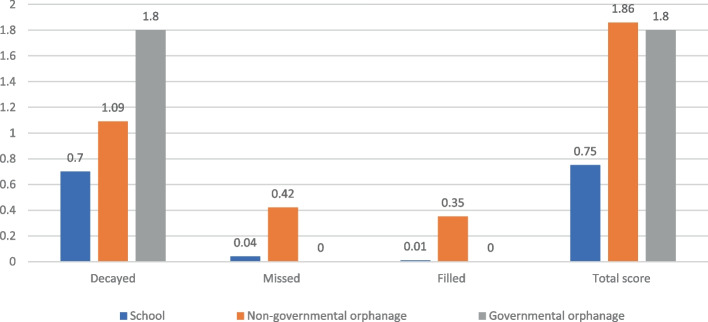
Mean DMF index components distribution among different study groups

**Fig. 5:**
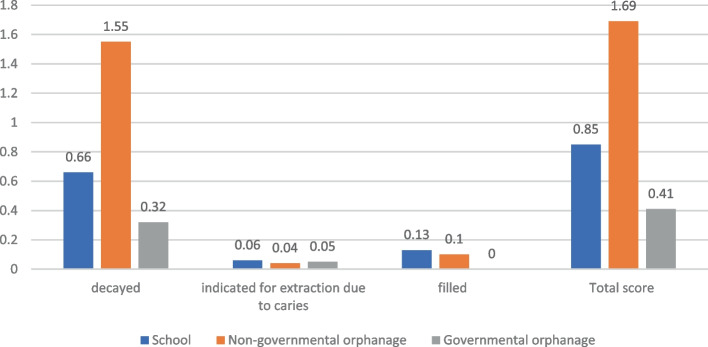
Mean def index components distribution among different study groups

The number and percentages of caries-free children were 11 (21.15%), 13 (25%), and 20 (38.46%) for governmental, non-governmental orphanages, and private primary school, respectively, while percentages of 41 (78.85%), 39 (75%), and 32 (61.54%) for governmental, non-governmental orphanages, and private primary school, respectively were found to have a previous caries experience, Fig. 3. The mean values for DMF/ def caries indices and their components among children with different dental caries experiences are demonstrated in Table 3, Figs. 4, 5.

**Table 3:** Mean and standard deviation values for DMF and def total scores and their components among different study groups

	**Private primary school** **(control group)**	**Non-governmental orphanage** **(Study group 1)**	**Governmental orphan**age **(Study group 2)**	*p*-value*
**DMF**				
Decayed (mean/SD)	0.7 (1.18)	1.09 (1.80)	1.80 (2.54)	0.561
Missed (mean/SD)	0.04 (0.31)	0.42 (1.10)	0 (0)	0.0001^d^
Filled (mean/SD)	0.01 (0.08)	0.35 (1.09)	0 (0)	0.0001^d^
Total score (mean/SD)	0.75 (1.29)	1.86 (2.96)	1.80 (2.54)	0.025^a^
**def**				
decayed (mean/SD)	0.66 (1.18)	1.55 (2.29)	0.32 (0.69)	0.006^b^
indicated for extraction due to caries (mean/SD)	0.06 (0.43)	0.04 (0.25)	0.05 (0.31)	0.931
filled (mean/SD)	0.13 (0.62)	0.10 (0.53)	0 (0)	0.906
Total score (mean/SD)	0.85 (1.79)	1.69 (2.58)	0.41(0.89)	0.01^c^

*p-value: Kruskal–Wallis H, ^a^*p* ≤ 0.05; ^b^*p* ≤ 0.01, ^c^*p* ≤ 0.001and ^d^*p* ≤ 0.0001

The highest significant caries index was recorded in the governmental orphanage (4.29), while the lowest mean was recorded in private primary school (2.17). The non-governmental orphanage showed a mean score of 2.5, Table 4. The restorative care index and the unmet treatment needs index are demonstrated in Table 5.

**Table 4:** Descriptive statistics and comparison of significant caries index between groups

	**Mean**	**SD**	**Minimum**	**Maximum**	**p-value**
Private primary school	2.17^b^	1.22	1	5	0.001
Non-governmental orphanage	2.5^b^	1.79	2	6	
Governmental orphanage	4.29^a^	2.97	2	14	

Significance level *P* < 0.05, *significant

Mann-Whitny U-test with Tukey’s post hoc test means sharing the same superscript test is not significantly different.

**Table 5:** The mean restorative care index and unmet treatment needs index for different study groups

Caries index	Private primary school (Control group)	Non-governmental orphanage (Study group 1)	Governmental Orphanage (Study group 2)
Restorative care index	1.33%	18.80%	0%
Unmet treatment needs index	93%	58.6%	100%

## Discussion

Dental caries is the most prevalent dental disease among school children and is considered a disease of childhood [29] and several studies were conducted to assess dental caries among different populations of children. Although orphans contribute to 2% of the world's population, and 1% of the Egyptian children population, literature regarding their dental health status, especially in Egypt, is vague [9, 30].

The children residing in orphanages are underprivileged and do not receive enough care as other children who live with their parents [10]. Several studies have examined the relationship between oral health and maternal deprivation and have found a positive association between both of them [31, 32].

To the fullest of our knowledge, few studies are comparing the dental caries experience in orphan institutionalized children to their counterparts parented children [13–16], and there is no previous study addressing the issue of oral health of orphans in Egypt. Since, health professionals, have an important role to play in tackling health disparities amongst their patients and more widely in the community [33], therefore, the present study was carried out to assess the dental caries status among two groups of institutionalized orphan children, resident in non-governmental and governmental orphanages, compared to a group of parented primary school children in Egypt. Orphan children were selected since there is growing recognition that the places where individuals live, learn, work, and play significantly influence their oral health, and addressing these social determinants of health in a community can lead to improved access to care [34].

The chosen children were in the age group range from six to 12 years old, which is a mixed dentition stage, so it was possible to assess caries experience in both primary and permanent dentition, also it was the most prevalent age among institutionalized orphans. Selected children were free from any systemic diseases or genetic disorders, including enamel/dentine defects that could influence their caries experience.

In Egypt, there are non-governmental orphanages and governmental orphanages, and this study was conducted on samples from both, to through light on the oral health status of both residents. A sample of children from a private primary school was taken as a comparator, as private schools attendees represent middle to high socioeconomic levels, and they have feasible accessibility. Permission to access the governmental schools was difficult to obtain, and the children attending these schools are subjected to poverty, which might be a confounder [35].

The examination was carried out following the WHO basic methods [23] to facilitate comparison with other previous studies that followed the same criteria and methods. The examination was conducted in a separate room on an ordinary chair at daylight, to standardize the examination protocol for all study populations. DMF [25] and def [26] indices were used for the assessment of dental caries, also the unmet treatment needs index, the restorative care index, as well as the significant caries index, were recorded.

The results of the current study revealed that the prevalence of dental caries and the mean values for both DMFT and deft were higher among orphan residents of both orphanages compared to school children Table [3], and Fig. 3). This matched the findings reported by Meshki et al., 2021,[13] who run their study in Iran and reported mean DMT total score and dmf total score of (3.36 ± 1.86, and 9.01 ± 3.8, respectively) in the orphan children compared to (2.1 ± 1.8, and 5.2 ± 3.4, respectively) in non-orphan children. Also, it goes in agreement with the results of Al-Jober et al., 2013,[14] who reported a prevalence of dental caries among children in Saudi Arabia (96%) compared to (90%) in the control group of children. On the other hand, the results of the current study contradict the results reported by Maweri et al., 2014, [15] study, who conducted their study in Yamen and reported a lower prevalence of caries and lower dmf total mean scores in the orphan children (84.7%, and 2.28 ± 2.37, respectively) in contrast of (89.6%, and 3.82 ± 2.57, respectively) in the non-orphan children, however, the mean DMF total scores were (2.06 ± 1.94, and 1.77 ± 1.58) in orphan children and non-orphan children, respectively. The difference in results could be attributed to difference in age groups of the target population; the majority of children in the previous study were in the age group of 12–15 years, which is the permanent dentition stage age range.

Also, Sinha et al., 2017, [16] reported a lower prevalence of dental caries in the permanent teeth among Indian orphan children (58.9%) than among school children (82.7%), however, the caries prevalence in the primary teeth was (66.4%, and 27.9%) in orphan children and school children, respectively. The contrast and agreement between the current study and the previous studies' findings could be owed to many determinants of oral health, such as demographics, social and economic factors, physical environment, clinical care, health behavior, and health outcomes that directly affect the dental caries prevalence and experience among different populations [34, 36].

Moreover, the present results revealed, a higher level of significant caries index among governmental orphanage children (4.29) than among non-governmental orphanage (2.5) and private primary school children (2.17). The higher prevalence of dental caries, higher dental caries experience score represented by DMFT and deft, and significant caries index among orphanages children in comparison to parented school children could be explained on the basis that the absence of family care, along with the awareness and perception of the caretakers in the orphanages, could influence the general and oral health behavior of the children, and consequently affects their caries experience negatively [2, 4, 37].

In Egypt, there are governmental-owed orphanages, which are administered and funded by the government, and non-governmental orphanages, which are run and administrated by nonprofit institutions of civil society. Unlike the non-governmental orphanages, the governmental orphanage's children in the current study displayed a lower level of dental caries experience, represented by the def and the DMF total scores. Despite the limited financial resources and access to dental care available for the governmental orphanage, they have a strict lifestyle for their orphan children residents, especially regarding dietary habits. This fact is reflected by the participants' answers to the questions regarding their brushing habits, snacking habits, and if they visited a dentist before, Table 2, and Fig. 2. The differences in the results between the non-governmental and the governmental orphanages in the current study could be justified based on these findings. These results go following Kamran et al., 2017 [38] who reported a highly significant association between the type of orphanage and DMF score, in which the governmental-owed orphanage recorded a higher prevalence than the private-owned orphanage.

The current results also revealed, the lowest restorative care index (0%) and the highest unmet treatment need index (100%) among the residents of governmental orphanage compared to the other study groups, which reflects limited access to professional dental care. These findings were in agreement with Kamran et al., 2017, who reported in their study that 66.2% of orphan children suffered from dental pain had not visited the dentist during the last year [38]. Despite, the higher caries experience of children living in the non-governmental orphanage, probably owing to poor oral hygiene and improper dietary habit, yet, they have the highest level of restorative care index and the lowest treatment needs compared to governmental orphanage children, which account for better access to professional dental care, provided by the civil community services and universities teaching hospitals.

In contrast to the non-governmental orphanage children, governmental orphanage children showed less caries experience score, owing to their restricted dietary habits. But yet, they have a lower restorative care index and higher treatment need index compared to parent children who have the advantage of having a responsible parent seeking dental care and treatment. The parented school children, however, only go to the dentist for symptomatic treatment, subsequently, although they have better care, yet, their unmet treatment needs index and restorative care index are still away from optimal. This conclusion is augmented by the findings of Helala et al., 2022, [39] who reported an inadequate practice of parents of a sample of Egyptian children towards their children's oral health (55.4%) with a strong negative statistically significant correlation between the parents' practice score and the total def score of their children (*r* = -0.55, and *p* = 0.0). The percentage of parents who correctly answered the question “when do you take your child to visit the dentist?” was reported to be (0%), which explains the inadequate restorative care index and high unmet treatment needs index in the parented children in the current study, compared to non-governmental orphanage children who have better access to the professional dental care.

The current study results revealed a higher caries prevalence among the governmental orphanage children compared to parented school children, although they have reported better tooth brushing habits and a lower intake of cariogenic snacks. The multifactorial nature of dental caries could explain these findings, since differences in tooth morphology and dental tissue composition, as well as salivary quality, quantity, and composition, could influence the occurrence of dental caries [40]. Moreover, insufficient supervision during teeth brushing among the orphanage children, due to the inappropriate care-taker to child ratio could be contributory factors to these findings [4].

The applicability of this study's results on other populations is expected to vary according to the demographic data and socioeconomic level of each orphan child, accessibility to professional dental care, oral health knowledge of the children and their caretakers, their oral hygiene and dietary habit practice, although the main hypothesis is expected to remain valid, as orphan children are underprivileged across different cultures and societies.

This study, although limited by the lack of assessment of children's comprehensive diet history analysis, oral hygiene status measurement, and oral health knowledge among children and their parents/ caretakers, yet reflected light on the demand of orphan children for regular professional dental care. However, this will only be controlled by the promotion of initiatives that prioritize the improvement in the social determinants of health as a backbone structure for the development of healthy enabling environments [41].

## Conclusions

Within the limitation of this case–control study and according to its findings, the institutionalized orphanage children had a higher prevalence of dental caries and dental caries experience compared to parented school children. Effective oral health preventive strategies, in addition to oral health educational programs targeting their caregivers, need to be implemented to improve the oral health status and oral health practices of those children. Also, enrollment of orphanage children in regular professional dental care programs is mandatory.

## Data Availability

Raw data (master table) is available upon request from the corresponding author.

## Abbreviation

ACU: Ahram Canadian University

## References

[1] Lisboa CM, de Paula JS, Ambrosano GMB, Pereira AC, Meneghim M de C, Cortellazzi KL, et al. Socioeconomic and family influences on dental treatment needs among Brazilian underprivileged schoolchildren participating in a dental health program. BMC Oral Health. 2013;13(1):56. Available from: https://bmcoralhealth.biomedcentral.com/articles/10.1186/1472-6831-13-56. 10.1186/1472-6831-13-56 PMC3854454 24138683

[2] Elshebani SB, Huew R, Buzaribah KS, Mansur EK (2022). Parental awareness and attitude about oral health habits of their children and its relation to caries experience in 8–10-year-old children. J Adv Educ Sci..

[3] Bozorgmehr E, Hajizamani A, Malek Mohammadi T. Oral Health Behavior of Parents as a Predictor of Oral Health Status of Their Children. ISRN Dent. 2013;2013:1–5. Available from: https://www.hindawi.com/journals/isrn/2013/741783/. 10.1155/2013/741783 PMC3664493 23738088

[4] Saleem J, Ishaq M, Butt MS, Zakar R, Malik U, Iqbal M, et al. Oral health perceptions and practices of caregivers at children’s religious schools and foster care centers: a qualitative exploratory study in Lahore, Pakistan. BMC Oral Health. 2022;22(1):641. Available from: https://bmcoralhealth.biomedcentral.com/articles/10.1186/s12903-022-02687-0. 10.1186/s12903-022-02687-0 PMC9789729 36566188

[5] Kitsaras G, Goodwin M, Kelly MP, Pretty IA. Bedtime Oral Hygiene Behaviours, Dietary Habits and Children’s Dental Health. Children. 2021;8(5):416. Available from: https://www.mdpi.com/2227-9067/8/5/416. 10.3390/children8050416 PMC8160840 34069504

[6] Naidu R, Nunn J, Forde M. Oral healthcare of preschool children in Trinidad: a qualitative study of parents and caregivers. BMC Oral Health. 2012;12(1):27. Available from: https://bmcoralhealth.biomedcentral.com/articles/10.1186/1472-6831-12-27. 10.1186/1472-6831-12-27 PMC3567990 22862892

[7] BaniHani A, Tahmassebi J, Zawaideh F. Maternal knowledge on early childhood caries and barriers to seek dental treatment in Jordan. Eur Arch Paediatr Dent. 2021;22(3):433–9. Available from: https://link.springer.com/10.1007/s40368-020-00576-0. 10.1007/s40368-020-00576-0 PMC8213663 33210223

[8] UNICEF. Factsheet: Children without primary caregivers and in institutions. Geneva: UNICEF; 2004.

[9] UNICEF. Africa's Orphaned and Vulnerable Generations: Children Affected by AIDS. Unicef; 2006.

[10] Kaur R, Vinnakota A, Panigrahi S, Manasa R V. A Descriptive Study on Behavioral and Emotional Problems in Orphans and Other Vulnerable Children Staying in Institutional Homes. Indian J Psychol Med. 2018;40(2):161–8. Available from: hhttp://journals.sagepub.com/doi/10.4103/IJPSYM.IJPSYM_316_17. 10.4103/IJPSYM.IJPSYM_316_17 PMC6008989 29962573

[11] Moffa M, Cronk R, Fejfar D, Dancausse S, Padilla LA, Bartram J. A systematic scoping review of hygiene behaviors and environmental health conditions in institutional care settings for orphaned and abandoned children. Sci Total Environ. 2019;658:1161–74. Available from: https://linkinghub.elsevier.com/retrieve/pii/S0048969718351593. 10.1016/j.scitotenv.2018.12.286 30677980

[12] Camacho GA, Camacho E, Rodríguez RA, de J Guillé A, Juárez HM, Pérez MG. Predisposing factors for dental caries in girls at an orphanage of Mexico City. Acta Pediatr Mex. 2009;30(2):71–6.

[13] Meshki R, Basir L, Motaghi S, Kazempour M. Oral health status among orphan and non-orphan children in Mashhad: a case-control study. J Med Life. 2022;15(9):1198–201. Available from: https://medandlife.org/wp-content/uploads/JMedLife-15-1198.pdf. 10.25122/jml-2021-0127 PMC9635224 36415523

[14] Al-Jobair AM, Al-Sadhan SA, Al-Faifi AA, Andijani RI. Medical and dental health status of orphan children in central Saudi Arabia. Saudi Med J. 2013;34(5):531–6. Available from: http://www.ncbi.nlm.nih.gov/pubmed/23677271. 23677271

[15] Al-Maweri S, Al-Soneidar W, Halboub E. Oral lesions and dental status among institutionalized orphans in Yemen: A matched case-control study. Contemp Clin Dent. 2014;5(1):81. Available from: http://www.contempclindent.org/text.asp?2014/5/1/81/128673. 10.4103/0976-237X.128673 PMC4012124 24808701

[16] Sinha A, Kaur K, Singh K, Puri MS, Anandani CKJ (2017). Dental Caries Status Among Orphans And Parented Children In North India: A Comparative Study. J Adv Med Dent Sci Res.

[17] Tomar SL. Planning and Evaluating Community Oral Health Programs. Dent Clin North Am. 2008;52(2):403–21. Available from: https://linkinghub.elsevier.com/retrieve/pii/S0011853207001061. 10.1016/j.cden.2007.11.005 18329451

[18] Jha K, Jalaluddin M, Suresan V, Diptajit D, Sourav S, Fatima A. Dental Caries Experience and Oral Hygiene Status among Institutionalized Orphans of Bhubaneswar City, Odisha: A Comprehensive Dental Healthcare Program Outcome. World J Dent. 2021;12(2):131–7. Available from: https://www.wjoud.com/doi/10.5005/jp-journals-10015-1810.

[19] Agrawal A, Rani P, Srilatha S, Khare V, Koshy A, Kapse SC. Prevalence of Dental Caries and Treatment Needs among the Orphan Children and Adolescents of Udaipur District, Rajasthan, India. J Contemp Dent Pract. 2012;13(2):182–7. Available from: https://www.thejcdp.com/doi/10.5005/jp-journals-10024-1118. 10.5005/jp-journals-10024-1118 22665745

[20] Tangade P, Batra M, Tirth A, Ravishankar T, Shah AF, Pal S. Dental Caries Status of Institutionalized Orphan Children from Jammu and Kashmir, India. Int J Clin Pediatr Dent. 2016;9(4):364–71. Available from: https://www.ijcpd.com/doi/10.5005/jp-journals-10005-1392. 10.5005/jp-journals-10005-1392 PMC5233705 28127170

[21] Solis-Riggioni A, Gallardo-Barquero C, Chavarria-Bolaños D. Prevalence and Severity of Dental Caries in Foster-Care Children and Adolescents. J Clin Pediatr Dent. 2018;42(4):269–72. Available from: https://meridian.allenpress.com/jcpd/article/42/4/269/78910/Prevalence-and-Severity-of-Dental-Caries-in. 10.17796/1053-4628-42.4.5 29750620

[22] von Elm E, Altman DG, Egger M, Pocock SJ, Gøtzsche PC, Vandenbroucke JP. The Strengthening the Reporting of Observational Studies in Epidemiology (STROBE) Statement: Guidelines for reporting observational studies. Int J Surg. 2014;12(12):1495–9. Available from: https://linkinghub.elsevier.com/retrieve/pii/S174391911400212X. 10.1016/j.ijsu.2014.07.013 25046131

[23] Organization WH. Oral health surveys: basic methods. World Health Organization; 2013.

[24] Organization WH. Oral Health Surveys: Basic Methods. Biometrics. 1971;27(4):1111. Available from: https://www.jstor.org/stable/2528861?origin=crossref.

[25] Klein H, Palmer CE, Knutson JW. Studies on Dental Caries: I. Dental Status and Dental Needs of Elementary School Children. Public Heal Reports. 1938;53(19):751. Available from: https://www.jstor.org/stable/10.2307/4582532?origin=crossref.

[26] Gruebbel AO. A Measurement of Dental Caries Prevalence and Treatment Service for Deciduous Teeth. J Dent Res. 1944;23(3):163–8. Available from: http://journals.sagepub.com/doi/10.1177/00220345440230030201.

[27] Walsh J (1970). International patterns of oral health care – the example of New Zaeland. NZ Dent J.

[28] Jong A. Dental Public Health and Community Dentistry. St. Louis: C.V. Mosby Co.; 1981. p. 74–88.

[29] Petersen PE, Bourgeois D, Ogawa H, Estupinan-Day S, Ndiaye C (2005). The global burden of oral diseases and risks to oral health. Bull World Health Organ..

[30] The Central Agency for Public Mobilization and Statistics (Egypt), and UNICEF Egypt. Children in Egypt: a statistical digest 2016. Cairo; 2017.

[31] Locker D. Deprivation and oral health: a review. Community Dent Oral Epidemiol. 2000;28(3):161–9. Available from: http://doi.wiley.com/10.1034/j.1600-0528.2000.280301.x. 10.1034/j.1600-0528.2000.280301.x 10830642

[32] Muirhead V, Marcenes W. An ecological study of caries experience, school performance and material deprivation in 5-year-old state primary school children. Community Dent Oral Epidemiol. 2004;32(4):265–70. Available from: https://onlinelibrary.wiley.com/doi/10.1111/j.1600-0528.2004.00147.x. 10.1111/j.1600-0528.2004.00147.x 15239777

[33] Williams DM, Sheiham A, Watt RG. Oral health professionals and social determinants. Br Dent J. 2013;214(9):427–427. Available from: http://www.nature.com/articles/sj.bdj.2013.436. 10.1038/sj.bdj.2013.436 23660879

[34] Chazin S, Glover J. A community framework for addressing social determinants of Oral health for low-income populations. Center for Health Care Strategies, Inc. 2017.

[35] Foley M, Akers H. Does poverty cause dental caries? Aust Dent J. 2018; Available from: https://onlinelibrary.wiley.com/doi/10.1111/adj.12666. 10.1111/adj.12666 30444538

[36] de Abreu MHNG, Cruz AJS, Borges-Oliveira AC, Martins R de C, Mattos F de F. Perspectives on Social and Environmental Determinants of Oral Health. Int J Environ Res Public Health. 2021;18(24):13429. Available from: https://www.mdpi.com/1660-4601/18/24/13429. 10.3390/ijerph182413429 PMC8708013 34949037

[37] Hooley M, Skouteris H, Boganin C, Satur J, Kilpatrick N. Parental influence and the development of dental caries in children aged 0–6 years: A systematic review of the literature. J Dent. 2012;40(11):873–85. Available from: https://linkinghub.elsevier.com/retrieve/pii/S030057121200200X. 10.1016/j.jdent.2012.07.013 22842202

[38] Kamran R, Farooq W, Faisal MR, Jahangir F. Clinical consequences of untreated dental caries assessed using PUFA index and its covariates in children residing in orphanages of Pakistan. BMC Oral Health. 2017;17(1):108. Available from: http://bmcoralhealth.biomedcentral.com/articles/10.1186/s12903-017-0399-9. 10.1186/s12903-017-0399-9 PMC5504620 28693477

[39] Helal M, Moneim SA, Foad M (2022). Parents' knowledge, attitude and practices toward oral health of their children with primary dentition: A cross sectional study. J Med Sci Res..

[40] Butera A, Maiorani C, Morandini A, Simonini M, Morittu S, Trombini J, et al. Evaluation of Children Caries Risk Factors: A Narrative Review of Nutritional Aspects, Oral Hygiene Habits, and Bacterial Alterations. Children. 2022;9(2):262. Available from: https://www.mdpi.com/2227-9067/9/2/262. 10.3390/children9020262 PMC8870668 35204983

[41] Tellez M, Zini A, Estupiñan-Day S. Social Determinants and Oral Health: An Update. Curr Oral Heal Reports. 2014;1(3):148–52. Available from: http://link.springer.com/10.1007/s40496-014-0019-6.

